# One Health Approach for Reporting Veterinary Carbapenem-Resistant Enterobacterales and Other Bacteria of Public Health Concern

**DOI:** 10.3201/eid2906.221648

**Published:** 2023-06

**Authors:** Kate KuKanich, Amy Burklund, Rob McGaughey, Nancy Muturi, Sasha Thomason, M.M. Chengappa, Ingrid Garrison, Bryna Stacey, Shuping Zhang, Tamara Gull

**Affiliations:** Kansas State University, Manhattan, Kansas, USA (K. KuKanich, A. Burklund, R. McGaughey, N. Muturi, S. Thomason, M.M. Chengappa);; Kansas Department of Health and Environment, Topeka, Kansas, USA (I. Garrison, B. Stacey);; University of Missouri, Columbia, Missouri, USA (S. Zhang, T. Gull)

**Keywords:** carbapenem-resistant Enterobacterales, One Health, carbapenem-resistant, companion animals, educational flyers, Enterobacterales, *Pseudomonas*, public health, *Staphylococcus aureus*, *Staphylococcus pseudintermedius*, antimicrobial resistance, bacteria, MRSA and other staphylococci, zoonoses, Kansas, Missouri, United States

## Abstract

A carbapenem-resistant Enterobacterales outbreak at a veterinary teaching hospital in the United States increased urgency for improved communication among diagnostic laboratories, public health authorities, veterinarians, and pet owners. Kansas State University, University of Missouri, Kansas Department of Health and Environment, and Veterinary Laboratory Investigation and Response Network created a surveillance, storage, and reporting protocol for veterinary antimicrobial-resistant bacteria; determined frequency of those bacteria in companion animals during 2018–2021; and created educational flyers for veterinarians and pet owners. We recommend a One Health strategy to create efficient surveillance programs to identify and report antimicrobial-resistant bacteria and educate veterinarians and pet owners about transmission risks.

Companion animals share living environments with their owners, creating opportunities for sharing bacteria (*1*–*9*). Bacteria sharing can have substantial public health consequences because of emerging antimicrobial-resistant (AMR) bacteria, such as carbapenem-resistant Enterobacterales (CRE), carbapenem-resistant *Pseudomonas aeruginosa* (CRPA), and methicillin-resistant *Staphylococcus*, which can cause severe human infections and limit options for antimicrobial therapy. The Centers for Disease Control and Prevention (CDC) considers CRE to be an urgent threat and both CRPA and methicillin-resistant *Staphylococcus* to be serious human health threats (*10*); carbapenemase-producing CRE are of highest clinical concern and warrant a public health response (*11*).

CRE bacteria have been isolated from pets, but the true prevalence is unknown (*12*–*14*). However, in 2020, the University of Pennsylvania School of Veterinary Medicine reported a cluster of canine and feline CRE cases (*15*); those cases brought awareness and urgency to One Health professionals to create veterinary laboratory and hospital protocols for CRE reporting and response to improve patient management and minimize transmission and public health effects.

Many states do not require reporting of specific AMR bacteria isolated from veterinary patients, yet the emergence of CRE and other AMR organisms in veterinary medicine has accelerated discussion of whether reporting should be required. Nationally, the Veterinary Laboratory Investigation and Response Network (Vet-LIRN) within the US Food and Drug Administration’s Center for Veterinary Medicine collaborates with laboratories in North America to perform veterinary AMR bacteria monitoring and can assist with further classification of mechanisms and outbreak investigations (*16*). Establishing a best practice protocol for internal laboratory tracking of AMR isolates and logistical case reporting to state public health authorities will enable efficient epidemiologic tracing and outbreak investigations, if needed. Furthermore, implementing a targeted response with educational material for veterinarians and pet owners will improve patient care and public health when AMR organisms are isolated from pets.

The goals of this study were to create a protocol to routinely surveil, store, and report CRE isolates and other bacteria of public health concern to state and national public health authorities; determine the prevalence of CRE, CRPA, and methicillin-resistant *Staphylococcus* in companion animals reported by the Kansas State Veterinary Diagnostic Laboratory (KSVDL) and University of Missouri Veterinary Medical Diagnostic Laboratory (MU-VMDL) during 2018–2021; and create educational flyers for veterinarians and pet owners that can be attached to bacteriology reports when CRE, CRPA, or methicillin-resistant *Staphylococcus* are found in companion animals, providing immediate access information, improved responses, and minimization of public health effects. A One Health approach was used in collaboration with veterinary and human healthcare professionals at local, state, and national agencies to create effective protocols and educational flyers that recognize and respond to public health concerns and reduce the risk of disease in animals and humans. We report the generation of protocols and flyers and the unique challenges associated with implementation encountered by KSVDL and MU-VMDL.

## Methods

We reviewed and streamlined the current KSVDL methods for storing AMR isolates. We created an efficient standard operating procedure to be applied within the KSVDL and MU-VMDL and made available at other diagnostic laboratories.

We reviewed regulations for reporting AMR organisms in Kansas and Missouri and created a prototype reporting form to enable sharing of pertinent data among the Kansas Department of Health and Environment (KDHE), Missouri Department of Health and Senior Services (MDHSS), and Vet-LIRN. We established optimal methods for identifying applicable isolates and routes of electronic report submission to KDHE and MDHSS.

We searched KSVDL and MU-VMDL records for all CRE, CRPA, and methicillin-resistant *Staphylococcus* isolates collected from any animal species during 2018–2021. We also collected total numbers of cultures with *Escherichia coli* or *Klebsiella*, *Proteus*, *Pseudomonas*, or *Staphylococcus* spp. growth for each host species. We summarized all data and performed descriptive statistical analyses. We did not use human participants or animals in our study; thus, ethical approval was not required.

We created 12 flyers to provide targeted education and improve veterinary response when AMR isolates were identified. We created separate flyers for CRE, CRPA, and methicillin-resistant *Staphylococcus* for distribution to small animal veterinarians, dog and cat owners, equid veterinarians, and horse owners. Flyer content for veterinarians provided information on bacterial transmission, infection prevention, patient management strategies, and public health considerations. Pet owner flyers were written using lay terms and included information about organisms and sources, guided owners to closely follow veterinarian recommendations, discussed transmission risks to humans, and provided precautions for the home, including cleaning suggestions. All flyers provided additional resource information. Flyer content was initially reviewed by microbiologists, veterinarians, infectious disease specialists, and epidemiologists at KDHE and Vet-LIRN, then reviewed for content, style, and distribution logistics by 3 regional general practice veterinarians. Flyers were reviewed by healthcare communication experts and a graphics designer, who edited and finalized the content.

## Results

### Protocol for Isolating and Storing CRE, CRPA, and Methicillin-Resistant *Staphylococcus*

KSVDL and MU-VMDL are accredited by the American Association of Veterinary Laboratory Diagnosticians and follow standard methods for bacteria isolation, identification, and antimicrobial susceptibility testing. Both laboratories use matrix-assisted laser desorption/ionization time-of-flight mass spectrometry (Biotyper; Bruker Corp., https://www.bruker.com) to identify bacteria. KSVDL reports the *S. intermedius* group for isolates that includes *S. pseudintermedius, S. intermedius*, and *S. delphini*. Because *S. pseudintermedius* is known to be the primary canine and feline pathogen in this group, *S. intermedius* group isolates reported by the KSVDL are interpreted by practicing veterinarians and referred to throughout this report as *S. pseudintermedius* (*17*,*18*). Both laboratories perform antimicrobial susceptibility tests by using Sensititre broth microwell dilution testing and provide MICs and interpretations in accordance with Clinical Laboratory Standards Institute standards (*19*).

Both laboratories have traditionally used imipenem as a representative carbapenem for bacteria susceptibility testing. Human breakpoints are used because no veterinary imipenem breakpoints are available (*19*). Imipenem breakpoints for Enterobacterales are <1 µg/mL for susceptible, 2 µg/mL for intermediate, and >4 µg/mL for resistant bacteria. For *Pseudomonas* spp., breakpoints are <2 µg/mL for susceptible, 4 µg/mL for intermediate, and >8 µg/mL for resistant bacteria. Further carbapenem testing is required to characterize *Proteus*, *Morganella*, and *Providencia* spp. as CRE because of intrinsically elevated MICs for imipenem (*11*). As of January 1, 2021, MU-VMDL began using both imipenem and meropenem as representative carbapenems for susceptibility testing; organisms showing phenotypic resistance to imipenem are tested further by using a meropenem Etest (bioMérieux, https://www.biomerieux.com). Meropenem breakpoints for Enterobacterales are <1 µg/mL for susceptible, 2 µg/mL for intermediate, and >4 µg/mL for resistant bacteria and, for *Pseudomonas aeruginosa*, <2 µg/mL for susceptible, 4 µg/mL for intermediate, and >8 µg/mL for resistant bacteria. No veterinary meropenem breakpoints are available; thus, human meropenem breakpoints are used (*20*). In 2021, KSVDL began sending imipenem-resistant isolates to MU-VMDL for meropenem testing. We excluded *Proteus*, *Morganella*, and *Providencia* spp. identified before 2021 that did not undergo meropenem testing from this study. Carbapenemase production was not analyzed at either laboratory.

KSVDL and MU-VMDL use oxacillin as their representative penicillinase-resistant β-lactam antimicrobial. Oxacillin breakpoints are <2 µg/mL for susceptible and >4 µg/mL for resistant *S. aureus* and <0.25 µg/mL for susceptible and >0.5 µg/mL for resistant *S. pseudintermedius* (*19*). In accordance with CDC guidelines, *S. aureus* isolates showing phenotypic oxacillin resistance are tested further by using cefoxitin disks (Hardy Diagnostics, https://www.hardydiagnostics.com) and the Kirby-Bauer disk diffusion method. According to CDC guidelines, cefoxitin disk diffusion testing uses breakpoints (for zones of inhibition) of >22 mm for susceptible and <21 mm for resistant *S. aureus*. Coagulase-positive non–*S*. *aureus* staphylococci do not require cefoxitin disk diffusion testing to be classified as methicillin resistant (*21*).

In both laboratories, new CRE, CRPA, and methicillin-resistant *Staphylococcus* isolates from any animal host species are recultured weekly and stored in cryogenic storage vials at −80°C for further testing. Cryogenic tubes are labeled with unique identifying numbers, animal species, specimen type, genus and species, date, and initials of the microbiologist who froze the isolate. After the tubes are labeled, a sterile loop is used to pick colonies, which are then immersed in CryoSaver, Brucella Broth with Glycerol (10%) and Beads solution (Hardy Diagnostics) in cryogenic tubes. The tubes are vortexed and left at room temperature for 15 min. A sterile plastic pipette is used to remove the liquid, and tubes are stored at −80°C.

### Recording and Reporting CRE, CRPA, and Methicillin-Resistant *Staphylococcus* Data

We created a prototype reporting spreadsheet for animal isolate submissions that includes the submitting laboratory, date culture was finalized, animal species, specimen type and source (e.g., swab from ear), testing method, species, antimicrobials tested, MIC, and MIC interpretation (Appendix). We maintained confidentiality by excluding names of veterinarians, clinics, pet owners, pets, and contact information.

On the first day of each month, a report of CRE, CRPA, and methicillin-resistant *Staphylococcus* isolated by KSVDL was electronically generated by using an electronic medical record system (VetView version 2.0.18, https://www.vetview.org) and no-code workflow software (Decisions 6.7, https://www.decisions.com) to search the KSVDL database; search criteria was imipenem-resistant organisms isolated from any animal species. At MU-VMDL, the Sensititre SWIN software (Thermo Fisher Scientific, https://www.thermofisher.com) database was updated manually by using custom reporting categories each time a methicillin-resistant *Staphylococcus* or CRE organism was detected. The SWIN database was searched monthly for both methicillin-resistant *Staphylococcus* and CRE; data describing isolates were exported as a .csv file (comma-separate values), and duplicate entries were removed. Both laboratories maintain a comprehensive internal spreadsheet of clearly labeled stored isolates that can be added onto if further testing is performed, such as additional susceptibility tests or whole-genome sequencing.

In Kansas, human diagnostic laboratories are required to report human isolates of carbapenem-resistant organisms and vancomycin-intermediate/resistant *S*. *aureus* to KDHE within 24 hours and send a bacterial isolate, clinical specimen, or nucleic acid from any carbapenem-resistant organism (*22*). In Missouri, human isolates of vancomycin-intermediate/resistant *S*. *aureus* are reportable within 24 hours, and nosocomial MRSA are reportable quarterly (*23*). Carbapenem-resistant *Enterobacter* spp., *E. coli*, and *Klebsiella* spp. from human hosts are reportable quarterly as aggregates in Missouri, but carbapenemase-producing CRE are reportable immediately (*24*). In Kansas and Missouri, reporting is not required if those AMR organisms are isolated from veterinary patients (*25*,*26*).

Before sharing monthly surveillance reports with public health authorities outside the KSVDL and MU-VMDL, accession numbers were removed to maintain confidentiality. Then, the KSVDL spreadsheet was emailed to the AMR bacteria epidemiology team at KDHE, and the KSVDL and MU-VMDL spreadsheets were emailed to Vet-LIRN. During 2018–2021, MDHSS did not accept reports of animal-derived CRE, CRPA, or methicillin-resistant *Staphylococcus*, so direct reporting to the state was not pursued. We implemented the designed protocol for storing and reporting CRE, CRPA, and methicillin-resistant *Staphylococcus* at the KSVDL and MU-VMDL in January 2021 and have continued using the protocol since then.

### Prevalence of AMR Organisms

Bacteria classified as CRE were sporadically isolated from companion animals during 2018–2021 (Appendix). Carbapenem-resistant *E. coli* was isolated from 2 canids by KSVDL (1 ear swab sample, 1 abscess sample) and from 6 animals by MU-VMDL (2 canine abdominal swab samples, 1 canine ear swab sample, 1 canine blood sample, 1 equine peritoneal fluid sample, and 1 equine wound sample). Carbapenem-resistant *E. coli* was found in 0.2% (2/1288) of *E. coli* isolates from canids at KSVDL, 0.2% (4/1705) of isolates from canines at MU-VMDL, and 0.6% (2/331) of isolates from equids at MU-VMDL. Carbapenem-resistant *K*. *pneumoniae* was isolated from 3 canids by KSVDL (2 urine samples, 1 wound sample) and from 2 canids by MU-VMDL (1 urine sample, 1 ear swab sample). Carbapenem-resistant *K*. *pneumoniae* was found in 1.5% (3/201) of all *Klebsiella* spp. isolates from canids at KSVDL; carbapenem-resistant *Klebsiella* spp. were found in 1% (4/409) of all *Klebsiella* spp. isolated at MU-VMDL.

In 2021, imipenem-resistant *Proteus* spp. were identified in 44.4% (55/124) of all *Proteus* spp. isolates from canids, 50% (1/2) of all *Proteus* spp. isolates from felids, and 50% (1/2) of all *Proteus* spp. isolates from equids at MU-VMDL; as well as in 3% (4/131) of all *Proteus* spp. isolates from canids and 29% (2/7) of *Proteus* spp. isolates from felids at KSVDL. All 57 *Proteus* spp. isolates from MU-VMDL were confirmed as meropenem-susceptible bacteria. Four imipenem-resistant *Proteus* spp. isolates from KSVDL (3 canine, 1 feline) were sent to MU-VMDL for meropenem testing, and all 4 were meropenem-susceptible bacteria.

**Figure 1:**
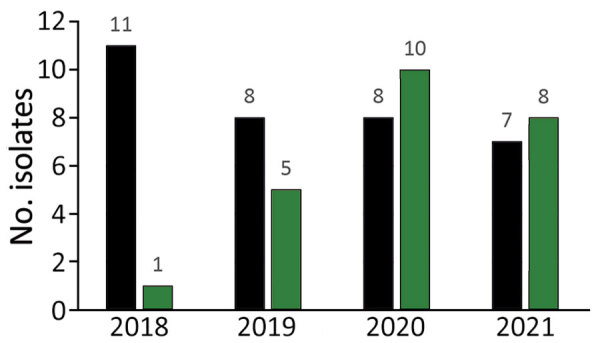
Numbers of carbapenem-resistant *Pseudomonas aeruginosa* isolates from canids in study of One Health approach for reporting veterinary carbapenem-resistant Enterobacterales and other bacteria of public health concern, United States, January 1, 2018–December 31, 2021. Black bars indicate isolates from Kansas State Veterinary Diagnostic Laboratory, green bars isolates from the University of Missouri Veterinary Medical Diagnostic Laboratory. At Kansas State Veterinary Diagnostic Laboratory, the total numbers of *P. aeruginosa* isolates from dogs were 159 in 2018, 150 in 2019, 146 in 2020, and 178 in 2021. At University of Missouri Veterinary Medical Diagnostic Laboratory, the total numbers of *P. aeruginosa* isolates from dogs were 151 in 2018, 169 in 2019, 166 in 2020, and 163 in 2021.

Carbapenem-resistant *Pseudomonas* spp. were isolated almost exclusively from dogs (Figure 1) but also from 2 cats and 1 horse. The numbers of CRPA isolates/total *Pseudomonas* spp. isolates from dogs were 11/159 (6.9%) in 2018, 8/150 (5.3%) in 2019, 8/146 (5.5%) in 2020, and 7/178 (3.9%) in 2021 at KSVDL. At MU-VMDL, CRPA isolates were identified in 1/151 (0.07%) dogs in 2018, 5/169 (3.0%) in 2019, 10/166 (6.0%) in 2020, and 8/163 (4.9%) in 2021. CRPA isolates from canids were collected from ear swab samples (n = 32), skin and wounds (n = 12), urogenital samples (n = 9), respiratory samples (n = 3), and unspecified sites (n = 2).

**Figure 2:**
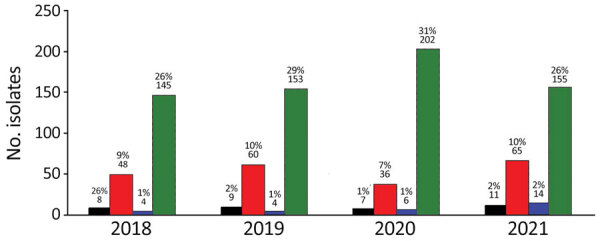
Numbers of MRSA and methicillin-resistant *Staphylococcus* *pseudintermedius* isolates from canids in study of One Health approach for reporting veterinary carbapenem-resistant Enterobacterales and other bacteria of public health concern, United States, January 1, 2018–December 31, 2021. Numbers and percentages of antimicrobial-resistant isolates (compared with all canine *Staphylococcus* spp. isolates) per year for each laboratory are shown. Kansas State Veterinary Diagnostic Laboratory isolates are indicated by black bars (MRSA) and green bars (methicillin-resistant *S*. *pseudintermedius*); University of Missouri Veterinary Medical Diagnostic Laboratory isolates are indicated by blue bars (MRSA) and green bars (methicillin-resistant *S*. *pseudintermedius*). Total numbers of *Staphylococcus* spp. from canids at Kansas State Veterinary Diagnostic Laboratory were 536 in 2018, 578 in 2019, 530 in 2020, and 680 in 2021. Total numbers of *Staphylococcus* spp. isolates from canids at University of Missouri Veterinary Medical Diagnostic Laboratory were 563 in 2018, 532 in 2019, 661 in 2020, and 608 in 2021.

**Figure 3:**
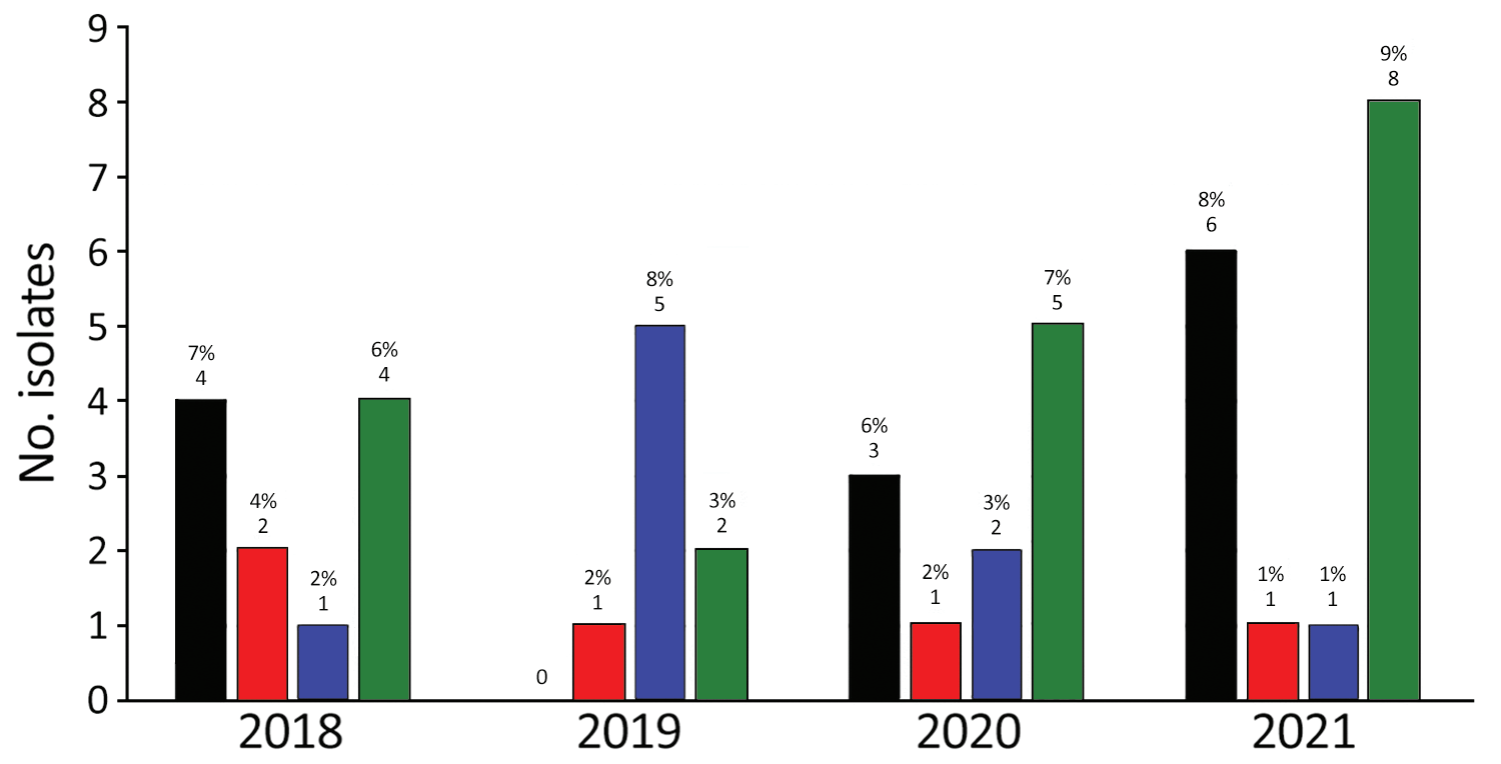
Numbers of MRSA and methicillin-resistant *Staphylococcus pseudintermedius* isolates from felids in study of One Health approach for reporting veterinary carbapenem-resistant Enterobacterales and other bacteria of public health concern, United Stated, January 1, 2018–December 31, 2021. Numbers and percentages of antimicrobial-resistant isolates (compared with all feline *Staphylococcus* spp. isolates) per year for each laboratory are shown. Kansas State Veterinary Diagnostic Laboratory isolates are indicated by black bars (MRSA) and red bars (methicillin-resistant *S*. *pseudintermedius*); University of Missouri Veterinary Medical Diagnostic Laboratory isolates are indicated by blue bars (MRSA) and green bars (methicillin-resistant *S*. *pseudintermedius*). Total numbers of *Staphylococcus* spp. isolates from felids at Kansas State Veterinary Diagnostic Laboratory were 54 in 2018, 60 in 2019, 55 in 2020, and 79 in 2021. Total numbers of *Staphylococcus* spp. isolates from felids at University of Missouri Veterinary Medical Diagnostic Laboratory were 63 in 2018, 60 in 2019, 67 in 2020, and 85 in 2021.

MRSA and methicillin-resistant *S*. *pseudintermedius* were isolated by KSVDL and MU-VMDL during 2018–2021 from both dogs and cats (Figures 2, 3). The most common sources of infection were skin (including abscesses, incisions, pyoderma, and wounds) in 34/63 dogs and 9/22 cats, urine (4/63 dogs, 6/22 cats), nose (6/63 dogs, 2/22 cats), ear (3/63 dogs, 3/22 cats), bones or joints (4/63 dogs, 0/22 cats), and the oral cavity (0/63 dogs, 1/22 cats); location was not reported for 15 dogs and 0 cats. Of 334 *Staphylococcus* spp. isolates from equids at MU-VMDL during 2018–2021, 5 were MRSA (1.5%) and 1 was methicillin-resistant *S*. *pseudintermedius* (0.3%). MRSA infections in equids were found in 3 wounds, 1 umbilicus sample, and 1 nasal sample; the sole equine methicillin-resistant *S*. *pseudintermedius* isolate was from an abscess. MRSA and methicillin-resistant *S*. *pseudintermedius* were not isolated from equine samples at KSVDL during 2018–2021. 

To directly communicate the public health importance of AMR isolation and help guide an optimal response, when a CRE, CRPA, or methicillin-resistant *Staphylococcus* spp. was isolated from a dog, cat, or horse by KSVDL or MU-VMDL, the corresponding veterinarian and pet owner flyers were digitally attached as .pdf (portable document format) files to the final bacteriology report sent to the submitting veterinarian. Those flyers could be downloaded, printed, and shared among veterinary staff and pet owners. The flyers were also posted online and available for download by the public (*27*–*29*). To attach flyers, KSVDL used the VetView electronic record system and Decisions 6.7 software, which can be programmed with search criteria, such as imipenem-resistant, *E. coli*, and dog; for all newly finalized bacteriology reports, flyers were attached automatically to those reports that fit the criteria. At MU-VMDL, VetView was used routinely; however, Decisions software was not available because of cost. Thus, isolates of interest were identified, and flyers were individually attached to each relevant bacteriology report by a microbiologist either digitally for email or manually for fax transmission.

## Discussion

CRE were only sporadically isolated at KSVDL and MU-VMDL during 2018–2021. However, in February 2022, a dog recently imported from Iran to the midwestern United States was confirmed to have New Delhi metallo-β-lactamase-5–producing carbapenem-resistant *E*. *coli*, and an additional 18 dogs in the same facility had confirmed CRE in rectal swab samples (*30*). Those cases reinforce that CRE are an urgent One Health threat. Carbapenem-resistant *E. coli* isolates from this study were not characterized further to identify carbapenemase production because they were isolated before storage protocols were in place. However, newly isolated CRE from KSVDL and UM-VMDL are now submitted to Vet-LIRN for whole-genome sequencing, when funding is available, to identify resistance genes (*31*,*32*). Although CRE prevalence from other US veterinary diagnostic laboratories is unknown and might differ from KSVDL and UM-VMDL, ongoing surveillance and educational efforts are recommended to guide veterinarians and protect pet owners.

MRSA was isolated infrequently from dogs and cats at both university laboratories; methicillin-resistant *S*. *pseudintermedius* was observed more frequently at MU-VMDL, likely because the laboratory receives a high case load of samples from 3 specialty dermatology practices. Both MRSA and methicillin-resistant *S*. *pseudintermedius* can cause infections of the skin, ears, surgical sites, and urinary tracts of dogs and cats but can also colonize pets without causing active infection. Furthermore, those bacteria are often resistant to many antimicrobial drugs, making treatment challenging (*33*). Methicillin-resistant *Staphylococcus* transmission is possible among companion animals, humans, and environmental reservoirs (*34*). Persons exposed to methicillin-resistant *S*. *pseudintermedius* tend to have rapid elimination without becoming clinically ill; however, immunocompromised persons can sometimes develop severe disease (*4*,*35*,*36*). MRSA is more often implicated in the transmission from humans to dogs, although transmission can occur in both directions (*6*,*37*). Clonal MRSA originating from humans has also been transmitted to horses, causing nosocomial equine infections (*2*), which places the horse, owner, rider, and veterinary personnel at risk for infection. Through surveillance and targeted educational flyers, One Health teams consisting of public health authorities, diagnostic laboratories, and veterinary staff can work together to recognize methicillin-resistant *Staphylococcus* and take actions to minimize further transmission at veterinary clinics and educate pet owners on minimizing transmission risk in the home and barn.

It is critical to use consistent definitions for AMR bacteria to enable clear communication and prevalence comparisons. Using imipenem as a representative carbapenem for susceptibility testing is a limitation at some veterinary diagnostic laboratories, including KSVDL; imipenem-resistant *Proteus* spp. isolated at KSVDL cannot be characterized as CRE unless further testing is performed (*38*). Laboratories using only imipenem should explore adding more in-house testing opportunities for *Proteus*, *Morganella*, and *Providencia* spp. or consider sending samples to laboratories with those testing capabilities. Carbapenems are rarely prescribed to companion animals and should be reserved for confirmed resistant infections in consultation with an infectious disease specialist, clinical veterinary pharmacologist, or microbiologist (*39*). Because clinical veterinarians might not have knowledge of carbapenem testing differences between Enterobacterales species, experts should provide advice and recommendations for additional susceptibility testing, when necessary, to obtain the most accurate susceptibility profile and determine optimal treatment strategies.

Financial resources are an additional challenge encountered by public health agencies monitoring AMR bacteria. Whereas most monitoring is passive and observational, preserving isolates and performing additional AMR bacteria susceptibility testing can be costly. In some cases, the pet owner will pay for additional testing to determine optimal therapy, but testing might also be requested for the sole purpose of improved understanding of regional AMR bacteria incidence. Additional funds can come from diagnostic laboratory or university administration, grant funds, or state public health funds. Dedication of resources (time, personnel, money) is vital for a successful AMR bacteria monitoring program.

Efficiently creating monthly reports of AMR veterinary isolates depends on having an automated electronic laboratory management system and knowledgeable information technology staff available to assist with setup, search parameters, and distribution of reports. At KSVDL, VetView and Decisions software enables creation of monthly excel spreadsheets that have targeted information for AMR bacteria and can be emailed to our research team automatically. However, not all electronic records systems are conducive to creating reports efficiently, and setup might be challenging, expensive, or require manual data collection. MU-VMDL used VetView as their primary record system, but they did not have the Decisions software available because of the additional cost; thus, identifying isolates and creating reports were more time-consuming.

When beginning a veterinary AMR bacteria surveillance and reporting program, who has access to AMR reports and what the intended uses will be should be clarified before reports are provided. Thus far, reports have been used for surveillance and regional prevalence determination of veterinary bacteria isolates and have created an opportunity for One Health discussions with state (KDHE) and federal (Vet-LIRN) public health teams about isolates of concern and additional testing needs. Furthermore, surveillance has enabled communication with veterinarians about those isolates through targeted educational flyers that can improve public health responses.

Collaborations between veterinary diagnostic laboratories and state public health authorities vary widely among US states. Kansas has an active state antimicrobial resistance program and an advisory group composed of statewide One Health professionals (*40*). Kansas State University veterinarians have an excellent working relationship with the state public health veterinarian and KDHE colleagues, and KDHE stays abreast of veterinary AMR isolates and collaborates on One Health research. In contrast, Missouri has been without a state public health veterinarian for several years, and MDHSS has not been able to invest in veterinary AMR surveillance. For diagnostic laboratories in states without a state public health veterinarian or those that do not have routine collaborative interaction with their state public health authorities, setting up a reporting system for AMR bacteria might be more challenging or less well received.

The primary limitation of our study is that the reach and effects of the educational flyers for veterinarians or pet owners were not measured. A future study could determine the basic level of knowledge of veterinarians regarding AMR organisms and measure knowledge learned or retained from flyers received for patient-specific situations. In addition, future studies could monitor rates of distribution of flyers to pet owners by veterinarians and determine the extent of public health knowledge and AMR transmission risk mitigation by pet owners.

In conclusion, we recommend a One Health strategy for creating an efficient surveillance program to identify and report regional bacterial isolates of concern and educating veterinarians and pet owners about transmission risks. The logistics of establishing such a program come with challenges that are unique to each laboratory. However, the benefits of increased awareness of isolate prevalence over time and having a specific plan for addressing positive cases will enable coordinated efforts that minimize effects of AMR organisms on veterinary patients and public health.

Appendix Additional information for One Health approach for reporting veterinary carbapenem-resistant Enterobacterales and other bacteria of public health concern.
